# Comparing Families of Dynamic Causal Models

**DOI:** 10.1371/journal.pcbi.1000709

**Published:** 2010-03-12

**Authors:** Will D. Penny, Klaas E. Stephan, Jean Daunizeau, Maria J. Rosa, Karl J. Friston, Thomas M. Schofield, Alex P. Leff

**Affiliations:** 1 Wellcome Trust Centre for Neuroimaging, University College, London, United Kingdom; 2 Branco-Weiss Laboratory for Social and Neural Systems Research, Empirical Research in Economics, University of Zurich, Zurich, Switzerland; Northwestern University, United States of America

## Abstract

Mathematical models of scientific data can be formally compared using Bayesian model evidence. Previous applications in the biological sciences have mainly focussed on model selection in which one first selects the model with the highest evidence and then makes inferences based on the parameters of that model. This “best model” approach is very useful but can become brittle if there are a large number of models to compare, and if different subjects use different models. To overcome this shortcoming we propose the combination of two further approaches: (i) family level inference and (ii) Bayesian model averaging within families. Family level inference removes uncertainty about aspects of model structure other than the characteristic of interest. For example: What are the inputs to the system? Is processing serial or parallel? Is it linear or nonlinear? Is it mediated by a single, crucial connection? We apply Bayesian model averaging within families to provide inferences about parameters that are independent of further assumptions about model structure. We illustrate the methods using Dynamic Causal Models of brain imaging data.

## Author Summary

Bayesian model comparison provides a formal method for evaluating different computational models in the biological sciences. Emerging application domains include dynamical models of neuronal and biochemical networks based on differential equations. Much previous work in this area has focussed on selecting the single best model. This approach is useful but can become brittle if there are a large number of models to compare and if different subjects use different models. This paper shows that these problems can be overcome with the use of Family Level Inference and Bayesian Model Averaging within model families.

## Introduction

Mathematical models of scientific data can be formally compared using Bayesian model evidence [1]–[3], an approach that is now widely used in statistics [4], signal processing [5], machine learning [6], natural language processing [7], and neuroimaging [8]–[10]. An emerging area of application is the evaluation of dynamical system models represented using differential equations, both in neuroimaging [11] and systems biology [12]–[14].

Much previous practice in these areas has focussed on model selection in which one first selects the model with the highest evidence and then makes inferences based on the parameters of that model [15]–[18]. This ‘best model’ approach is very useful but, as we shall see, can become brittle if there are a large number of models to compare, or if in the analysis of data from a group of subjects, different subjects use different models (as is the case for a random effects analysis [19]). This brittleness, refers to the fact that which is the best model can depend critically on which set of models are being compared. In random effects analysis, augmenting the comparison set with a single extra model can, for example, reverse the ranking of the best and second best models. To address this issue we propose the combination of two further approaches (i) family level inference and (ii) Bayesian model averaging within families.

**Figure 1:**
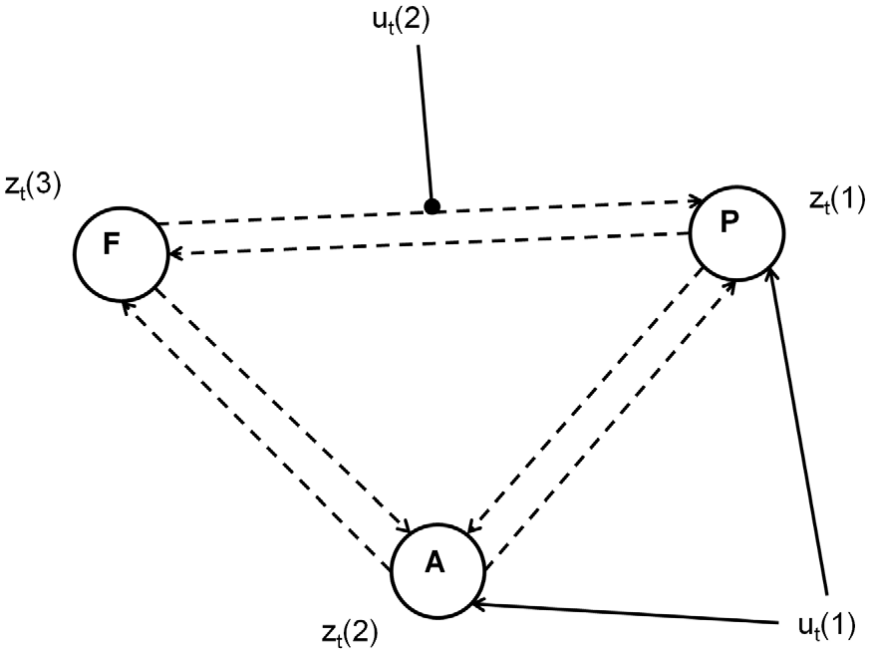
Dynamic Causal Models. The DCMs in this paper were used to analyse fMRI data from three brain regions: (i) left posterior temporal sulcus (region P), (ii) left anterior superior temporal sulcus (region A) and (iii) pars orbitalis of the inferior frontal gyrus (region F). The DCMs themselves comprised the following variables; experimental inputs 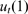 for auditory stimulation and 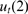 for speech intelligibility, a neuronal activity vector 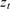 with three elements (one for each region P, A, and F), exogenous connections specified by the three-by-three connectivity matrix 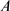 (dotted arrows in figure), modulatory connections specified by three-by-three modulatory matrics 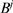 for inputs 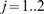 (the solid line ending with a filled circle denotes the single non-zero entry for this particular model), and a 3-by-2 direct input connectivity matrix 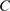 with non-zero entries shown by solid arrows. The dynamics of this model are govenered by equation 1. All DCMs in this paper used all-to-all endogenous connectivity ie. there were endogenous connections between all three regions. Different models were set up by specifying which regions received direct (auditory) input (non-zero entries in 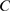) and which connections could be modulated by the speech intelligibility (non-zero entries in the matrix 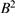).

We envisage that these methods will be useful for the comparison of large numbers of models (eg. tens, hundreds or thousands). In the context of neuroimaging, for example, inferences about changes in brain connectivity can be made using Dynamic Causal Models [20],[21]. These are differential equation models which relate neuronal activity in different brain areas using a dynamical systems approach. One can then ask a number of generic questions. For example: Is processing serial or parallel? Is it linear or nonlinear? Is it mediated by changes in forward or backward connections? A schematic of a DCM used in this paper is shown in Figure 1. The particular questions we will address in this paper are (i) which regions receive driving input? and (ii) which connections are modulated by other experimental factors?

This paper proposes that the above questions are best answered by ‘Family level inference’. That is inference at the level of model families, rather than at the level of the individual models themselves. As a simple example, in previous work [19] we have considered comparison of a number of DCMs, half of which embodied linear hemodynamics and half nonlinear hemodynamics. The model space was thus partitioned into two families; linear and nonlinear. One can compute the relative evidence of the two model families to answer the question: does my imaging data provide evidence in favour of linear versus nonlinear hemodynamics? This effectively removes uncertainty about aspects of model structure other than the characteristic of interest.

We have provided a simple illustration of this approach in previous work [19]. We now provide a formal introduction to family level inference and describe the key issues. These include, importantly, the issue of how to deal with families that do not contain the same number of models. Additionally, this paper shows how Bayesian model averaging can be used to provide a summary measure of likely parameter values for each model family. We provide an example of family-level inference using data from neuroimaging, a DCM study of auditory word processing, but envisage that the methods can be applied throughout the biological sciences. Before proceeding we note that the use of Bayesian model averaging is a standard approach in the field of Bayesian statistics [4], but has yet to be applied extensively in computational biology. The use of model families is also accomodated naturally within the framework of hierarchical Bayesian models [1] and is proposed to address the well known issue of model dilution [4].

## Materials and Methods

This section first briefly reviews DCM and methods for computing the model evidence. We then review the fixed and random effects methods for group level model inference, which differ as to whether or not subjects are thought to use the same or a different model. This includes the description of a novel Gibbs sampling method for random effects model inference that is useful when there are many models to compare. We then show that, for random effects inference, the selection of the single best model can be critically dependent on the set of models that are to be compared. This then motivates the subsequent subsection on family level inference, in which inferences about model characteristics are invariant to the comparison set. We describe family level inference in both a fixed and random effects context. The final subsection then describes a sample-based algorithm for implementing Bayesian model averaging using the notion of model families.

### Dynamic Causal Models

Dynamic Causal Modelling is a framework for fitting differential equation models of neuronal activity to brain imaging data using Bayesian inference. The DCM approach can be applied to functional Magnetic Resonance Imaging (fMRI), Electroencephalographic (EEG), Magnetoencephalographic (MEG), and Local Field Potential (LFP) data [22]. The empirical work in this paper uses DCM for fMRI. DCMs for fMRI comprise a bilinear model for the neurodynamics and an extended Balloon model [23] for the hemodynamics. The neurodynamics are described by the following multivariate differential equation
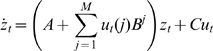
(1)where 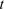 indexes continuous time and the dot notation denotes a time derivative. The 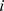
th entry in 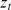 corresponds to neuronal activity in the 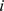
th region, and 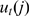 is the 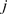
th experimental input.

A DCM is characterised by a set of ‘exogenous connections’, 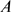, that specify which regions are connected and whether these connections are unidirectional or bidirectional. We also define a set of input connections, 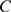, that specify which inputs are connected to which regions, and a set of modulatory connections, 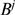, that specify which intrinsic connections can be changed by which inputs. The overall specification of input, intrinsic and modulatory connectivity comprise our assumptions about model structure. This in turn represents a scientific hypothesis about the structure of the large-scale neuronal network mediating the underlying cognitive function. A schematic of a DCM is shown in Figure 1.

In DCM, neuronal activity gives rise to fMRI activity by a dynamic process described by an extended Balloon model [24] for each region. This specifies how changes in neuronal activity give rise to changes in blood oxygenation that are measured with fMRI. It involves a set of hemodynamic state variables, state equations and hemodynamic parameters, 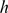. In brief, for the 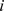
th region, neuronal activity 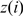 causes an increase in vasodilatory signal 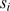 that is subject to autoregulatory feedback. Inflow 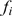 responds in proportion to this signal with concomitant changes in blood volume 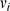 and deoxyhemoglobin content 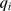.
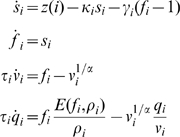
(2)Outflow is related to volume 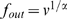 through Grubb's exponent 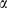 [20]. The oxygen extraction is a function of flow 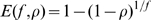 where 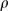 is resting oxygen extraction fraction. The Blood Oxygenation Level Dependent (BOLD) signal is then taken to be a static nonlinear function of volume and deoxyhemoglobin that comprises a volume-weighted sum of extra- and intra-vascular signals [20]

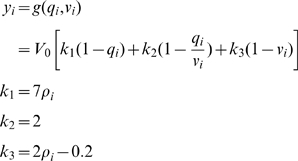
(3)where 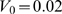 is resting blood volume fraction. The hemodynamic parameters comprise 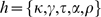 and are specific to each brain region. Together these equations describe a nonlinear hemodynamic process that converts neuronal activity in the 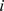 th region 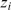 to the fMRI signal 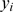 (which is additionally corrupted by additive Gaussian noise). Full details are given in [20],[23].

In DCM, model parameters 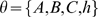 are estimated using Bayesian methods. Usually, the 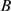 parameters are of greatest interest as these describe how connections between brain regions are dependent on experimental manipulations. For a given DCM indexed by 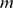, a prior distribution, 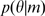 is specified using biophysical and dynamic constraints [20]. The likelihood, 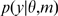 can be computed by numerically integrating the neurodynamic (equation 1) and hemodynamic processes (equation 2). The posterior density 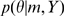 is then estimated using a nonlinear variational approach described in [23],[25]. Other Bayesian estimation algorithms can, of course, be used to approximate the posterior density. Reassuringly, posterior confidence regions found using the nonlinear variational approach have been found to be very similar to those obtained using a computationally more expensive sample-based algorithm [26].

### Model Evidence

This section reviews methods for computing the evidence for a model, 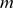, fitted to a single data set 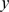. Bayesian estimation provides estimates of two quantities. The first is the posterior distribution over model parameters 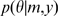 which can be used to make inferences about model parameters 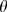. The second is the probability of the data given the model, otherwise known as the model evidence. In general, the model evidence is not straightforward to compute, since this computation involves integrating out the dependence on model parameters
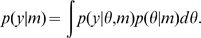
(4)


A common technique for approximating the above integral is the Variational Bayes (VB) approach [27]. This is an analytic method that can be formulated by analogy with statistical physics as a gradient ascent on the ‘negative variational Free Energy’ (or Free Energy for short), 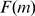, of the system. This quantity is related to the model evidence by the relation [27],[28]

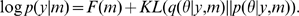
(5)where the last term in Eq.(5) is the Kullback-Leibler (KL) divergence between an ‘approximate’ posterior density, 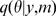, and the true posterior, 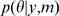. This quantity is always positive, or zero when the densities are identical, and therefore 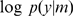 is bounded below by 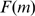. Because the evidence is fixed (but unknown), maximising 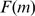 implicitly minimises the KL divergence. The Free Energy then becomes an increasingly tighter lower bound on the desired log-model evidence. Under the assumption that this bound is tight, model comparison can then proceed using 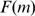 as a surrogate for the log-model evidence.

The Free Energy is but one approximation to the model evidence, albeit one that is widely used in neuroimaging [29],[30]. A simpler approximation, the Bayesian Information Criterion (BIC) [11], uses a fixed complexity penalty for each parameter. This is to be compared with the free energy approach in which the complexity penalty is given by the KL-divergence between the prior and approximate posterior [11]. This allows parameters to be differentially penalised. If, for example, a parameter is unchanged from its prior, there will be no penalty. This adaptability makes the Free Energy a better approximation to the model evidence, as has been shown empirically [6],[31].

There are also a number of sample-based approximations to the model evidence. For models with small numbers of parameters the Posterior Harmomic Mean provides a good approximation. This has been used in neuroscience applications, for example, to infer based on spike data whether neurons are responsive to particular features, and if so what form the dependence takes [32]. For models with a larger number of parameters the evidence can be well approximated using Annealed Importance Sampling (AIS) [33]. In a comparison of sample-based methods using synthetic data from biochemical networks, AIS provided the best balance between accuracy and computation time [13]. In other comparisons, based on simulation of graphical model structures [6] the Free Energy method approached the performance of AIS and clearly outperformed BIC. In this paper model evidence is approximated using the Free Energy.

### Fixed Effects Analysis

Neuroimaging data sets usually comprise data from multiple subjects as the perhaps subtle cognitive effects one is interested in are often only manifest at the group level. In this and following sections we therefore consider group model inference where we fit models 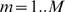 to data from subjects 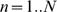. Every model is fitted to every subjects data. In Fixed Effects (FFX) Analysis it is assumed that every subject uses the same model, whereas Random Effects (RFX) Analysis allows for the possibility that different subjects use different models. This section focusses on FFX.

Given that our overall data set, 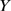, which comprises data for each subject, 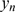, is independent over subjects, we can write the overall model evidence as
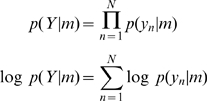
(6)Bayesian inference at the model level can then be implemented using Bayes rule
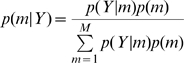
(7)Under uniform model priors, 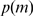, the comparison of a pair of models, 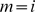 and 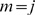, can be implemented using the Bayes Factor which is defined as the ratio of model evidences
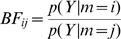
(8)Given only two models and uniform priors, the posterior model probability is greater than 0.95 if the BF is greater than twenty. Bayes Factors have also been stratified into different ranges deemed to correspond to different strengths of evidence. ‘Strong’ evidence, for example, corresponds to a BF of over twenty [34]. Under non-uniform priors, pairs of models can be compared using Odds Ratios. The prior and posterior Odds Ratios are defined as
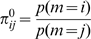
(9)

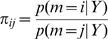
resepectively, and are related by the Bayes Factor
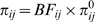
(10)When comparing two models across a group of subjects, one can multiply the individual Bayes factors (or exponentiate the sum of log evidence differences); this is referred to as the Group Bayes Factor (GBF) [16]. As is made clear in [19] the GBF approach implicitly assumes that every subject uses the same model. It is therefore a Fixed Effects analysis. If one believes that the optimal model structure is identical across subjects, then an FFX approach is entirely valid. This assumption is warranted when studying a basic physiological mechanism that is unlikely to vary across subjects, such as the role of forward and backward connections in visual processing [35].

### Random Effects Analysis

An alternative procedure for group level model inference allows for the possibility that different subjects use different models. This may be the case in neuroimaging when investigating pathophysiological mechanisms in a spectrum disease or when dealing with cognitive tasks that can be performed with different strategies. RFX inference is based on the characteristics of the population from which the subjects are drawn. Given a candidate set of 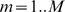 models, we denote 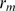 as the frequency with which model 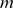 is used in the population. We also refer to 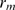 as the model probability.

We define a prior distribution over 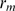 which in this paper, and in previous work [19], is taken to be a Dirichlet density (but see later)
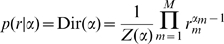
(11)where 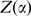 is a normalisation term and the parameters, 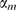, are strictly positively valued and can be interpreted as the number of times model 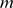 has been observed or selected. For 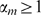 the density is convex in 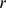-space, whereas for 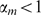 it is concave.

Given that we have drawn 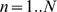 subjects from the population of interest we then define the indicator variable 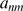 as equal to unity if model 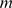 has been assigned to subject 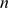. The probability of the ‘assignation vector’, 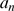, is then given by the multinomial density
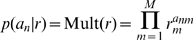
(12)The model evidence, 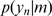, together with the above densities for model probabilities and model assignations constitutes a generative model for the data, 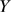 (see figure 1 in [19]). This model, can then be inverted to make inferences about the model probabilites from experimental data. Such an inversion has been described in previous work, which developed an approximate inference procedure based on a variational approximation [19] (this was in addition to the variational approximation used to compute the Free Energy for each model). The robustness and accuracy of this method was verified via simulations using data from synthetic populations with known frequencies of competing models [19]. This algorithm produces an approximation to the posterior density 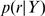 on which subsequent RFX inferences are based.

As we shall see in the following section, unbiased family level inferences require uniform priors over families. This requires that the prior model counts, 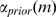, take on very small values (see equation 24). These values become smaller as the number of models in a family increases. It turns out that although the variational algorithm is robust for 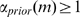, it is not accurate for 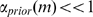. This is a generic problem with the VB approach and is explained further in the the supporting material (see file Text S1). For this reason, in this paper we choose to take a Gibbs sampling instead of a VB approach. Additionally, the use of Gibbs sampling allows us to relax the assumption made in VB that the posterior densities over 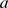 and 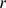 factorise [19]. Gibbs sampling is the Monte-Carlo method of choice when it is possible to iteratively sample from the conditional posteriors [1]. Fortunately, this is the case with the RFX models as we can iterate between sampling from 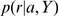 and 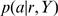. Such iterated sampling eventually produces samples from the marginal posteriors 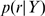 and 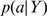 by allowing for a sufficient burn-in period after which the Markov-chain will have converged [1]. The procedure is described in the following section.

#### Gibbs sampling for random effects inference over models

First, model probabilites are drawn from the prior distribution
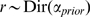
(13)where by default we set 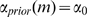 for all 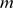 (but see later). For each subject 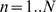 and model 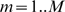 we use the model evidences from model inversion to compute
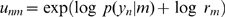
(14)

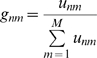
Here, 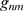 is our posterior belief that model 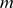 generated the data from subject 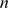 (these posteriors will be used later for Bayesian model averaging). For each subject, model assignation vectors are then drawn from the multinomial distribution
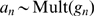
(15)We then compute new model counts
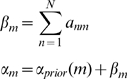
(16)and draw new model probabilities
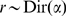
(17)Equations 14 to 17 are then iterated 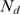 times. For the results in this paper we used a total of 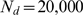 samples and discarded the first 10,000. These remaining samples then constitute our approximation to the posterior distribution 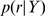. From this density we can compute usual quantities such as the posterior expectation, denoted 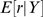 or 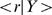. This completes the description of model level inference.

The above algorithm was derived for Dirichlet priors over model probabilities (see equation 11). The motivation for the Dirichlet form originally derived from the use of a free-form VB approximation [27] in which the optimal form for the approximate posterior density over 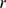 would be a Dirichlet if the prior over 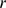 was also a Dirichlet. This is not a concern in the context of Gibbs sampling. In principle any prior density over 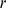 will do, but for continuity with previous work we follow the Dirichlet approach.

We end this section by noting that the Gibbs sampling method is to be preferred over the VB implementation for model level inferences in which the number of models exceeds the number of subjects, 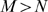. This is because it is important that the total prior count, 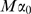, does not dominate over the number of subjects, otherwise posterior densities will be dominated by the prior rather than the data. This is satisfied, for example, by 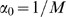. However, as described in the the supporting material (see file Text S1), the VB implementation does not work well for small 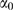. But if we wish to compare a small number of models then VB is the preferred method because it is faster as well as being accurate, as shown in previous simulations [19].

### Comparison Set

We have so far described procedures for Bayesian inference over models 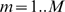. These models comprise the comparison set, 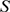. This section points out a number of generic features of Bayesian model comparison.

First, for any data set there exists an infinite number of possible models that could explain it. The purpose of model comparison is not to discover a ‘true’ model, but to determine that model, given a set of plausible alternatives, which is most ‘useful’, ie. represents an optimal balance between accuracy and complexity. In other words Bayesian model inference has nothing to say about ‘true’ models. All that it provides is an inference about which is more likely, given the data, among a set of candidate models.

Second, we emphasise that posterior model probabilities depend on the comparison set. For FFX inference this can be clearly seen in equation 7 where the denominator is given by a sum over 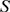. Similarly, for RFX inference, the dependence of posterior model probabilities on the comparison set can be seen in equation 14. Other factors being constant, posterior model probabilities are therefore likely to be smaller for larger 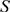.

Our third point relates to the ranking of models. For FFX analysis the relative ranking of a pair of models is not dependent on 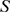. That is, if 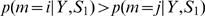 then 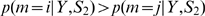 for any two comparison sets 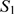 and 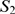 that contain models 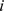 and 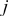. This follows trivially from equation 7 as the comparison set acts only as a normalisation term.

However, for group random effects inference the ranking of models can be critically dependent on the comparison set. That is, if 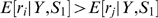 then it could be that 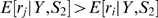 where 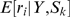 is the posterior expected probability of model 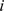 given comparison set 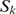. The same holds for other quantities derived from the posterior over 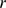, such as the exceedance probability (see [19] and later). This means that the decision as to which is the best model depends on 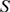. This property arises because different subjects can use different models and we illustrate it with the following example.

Consider that 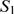 comprises just two models 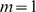 and 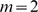. Further assume that we have 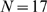 subjects and model 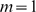 is preferred by 7 of these subjects and 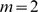 by the remaining 10. We assume, for simplicity, that the degrees of preference (ie differences in evidence) are the same for each subject. The quantity 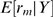 then simply reflects the proportion of subjects that prefer model 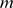 [19]. So 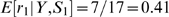, 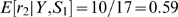 and for comparison set 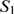 model 2 is the highest ranked model. Although the differences in posterior expected values are small the corresponding differences in exceedance probabilities will be much greater. Now consider a new comparison set 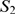 that contains an addditional model 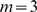. This model is very *similar* to model 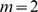 such that, of the ten subjects who previously preferred it, six still do but four now prefer model 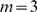. Again, assuming identical degrees of preference, we now have 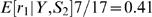, 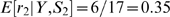 and 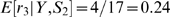. So, for comparison set 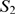 model 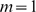 is now the best model. So which is the best model: model one or two?

We suggest that this seeming paradox shows, not that group random effects inference is unreliable, but that it is not always appropriate to ask which is the best model. As is usual in Bayesian inference it is wise to consider the full posterior density rather than just the single maximum posterior value. We can ask what is common to models two and three. Perhaps they share some structural assumption such as the existence of certain connections or other characteristic such as nonlinearity. If one were to group the models based on this characteristic then the inference *about the characteristic* would be robust. This notion of grouping models together is formalised using family-level inference which is described in the following section. One can then ask: of the models that have this characteristic what are the typical parameter values? This can be addressed using Bayesian Model Averaging within families.

### Family Inference

To implement family level inference one must specify which models belong to which families. This amounts to specifying a partition, 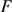, which splits S into 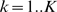 disjoint subsets. The subset 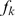 contains all models belonging family 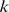 and there are 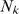 models in the 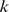 th subset.

Different questions can be asked by specifying different partitions. For example, to test model space for the ‘effect of linearity’ one would specify a partition into linear and nonlinear subsets. One could then test the same model space for the ‘effect of seriality’ using a different partition comprising serial and parallel subsets. The subsets must be non-overlapping and their union must be equal to S. For example, when testing for effects of “seriality”, some models may be neither serial or parallel; these models would then define a third subset.

The usefulness of the approach is that many models (perhaps all models) are used to answer (perhaps) all questions. This is similar to factorial experimental designs in psychology [36] where data from all cells are used to assess the strength of main effects and interactions. We now relate the two-levels of inference: family and model.

#### Fixed effects

To avoid any unwanted bias in our inference we wish to have a uniform prior at the family level
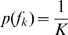
(18)Given that this is related to the model level as
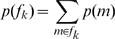
(19)the uniform family prior can be implemented by setting
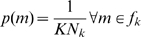
(20)The posterior distribution over families is then given by summing up the relevant posterior model probabilities
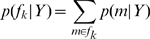
(21)where the posterior over models is given by equation 7. Because posterior probabilities can be very close to unity we will sometimes quote one minus the posterior probability. This is the combined probability of the alternative hypotheses which we refer to as the alternative probability, 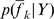.

#### Random effects

The family probabilities are given by
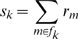
(22)where 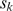 is the frequency of the family of models in the population. We define a prior distribution over this probability using a Dirichlet density
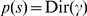
(23)A uniform prior over family probabilities can be obtained by setting 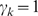 for all 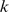. From equations 13 and 22 we see that this can be achieved by setting
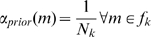
(24)We can then run the Gibbs sampling method described above for drawing samples from the posterior density 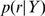. Samples from the family probability posterior, 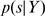, can then be computed using equation 22.

The posterior means, 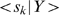, are readily computed from these samples. Another option is to compute an exceedance probability, 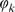, which corresponds to the belief that family 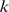 is more likely than any other (of the 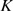 families compared), given the data from all subjects:
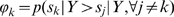
(25)Exceedance probabilities are particularly intuitive when comparing just two families as they can be written:
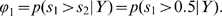
(26)


Family level inference addresses the issue of ‘dilution’ in model selection [4]. If one uses uniform model priors and many models are similar, then excessive prior probability is allocated to this set of similar models. One way of avoiding this problem is to use priors which dilute the probability within subsets of similar models ([4]). Grouping models into families, and setting model priors according to eg. equation 24, achieves exactly this.

### Bayesian Model Averaging

So far, we have dealt with inference on model-space, using partitions into families. We now consider inference on parameters. Usually, the key inference is on models, while the maximum a posteriori (MAP) estimates of parameters are reported to provide a quantitative interpretation of the best model (or family). Alternatively, people sometimes use subject-specific MAP estimates as summary statistics for classical inference at the group level. These applications require only a point (MAP) estimate. However for completeness, we now describe how to access the full posterior density on parameters, from which MAP estimates can be harvested.

The basic idea here is to use Bayesian model averaging within a family; in other words, summarise family-specific coupling parameters in a way that avoids brittle assumptions about any particular model. For example, the marginal posterior for subject 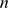 and family 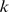 is
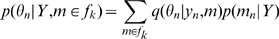
(27)where 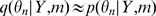 is our variational approximation to the subject specific posterior and 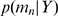 is the posterior probability that subject 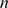 uses model 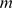. We could take this to be 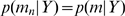 under the FFX assumption that all subjects use the same model, or 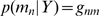 under the RFX assumption that each subject uses their own model (see equation 14).

Finally, to provide a single posterior density over subjects one can define the parameters for an average subject
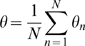
(28)and compute the posterior density 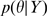 from the above relation and the individual subject posteriors from equation 27.

Equation 27 arises from a straightforward application of probability theory in which a marginal probability is computed by marginalising over quantities one is uninterested in (see also equation 4 for marginalising over parameters). Use of equation 27 in this context is known as Bayesian Model Averaging (BMA) [4],[37]. In neuroimaging BMA has previously been used for source reconstruction of MEG and EEG data [9]. We stress that no additional assumptions are required to implement equation 27.

One can make 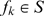 small or large. If we make 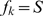, the entire model-space, the posteriors on the parameters become conventional Bayesian model averages where 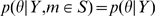. Conversely, if we make 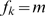, a single model, we get conventional parameter inference of the sort used when selecting the best model; i.e., 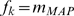. This is formally identical to using 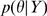 under the assumption that the posterior model density is a point mass at 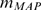. More generally, we want to average within families of similar models that have been identified by inference on families.

One can see from equation 27 that models with low probability contribute little to the estimate of the marginal density. This property can be made use of to speed up the implementation of BMA by excluding low probability models from the summation. This can be implemented by including only models for which
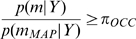
(29)where 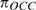 is the minimal posterior odds ratio. Models satisfying this criterion are said to be in Occam's window [38]. The number of models in the window, 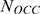, is a useful indicator as smaller values correspond to peakier posteriors. In this paper we use 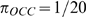. We emphasise that the use of Occam's window is for computational expedience only.

Although it is fairly simple to compute the MAP estimates of the Bayesian parameter (MAP) averages analytically, the full posteriors per se have a complicated form. This is because they are mixtures of Gaussians (and delta functions for models where some parameters are precluded a priori). This means the posteriors can be multimodal and are most simply evaluated by sampling. The sampling approach can be implemented as follows. This generates 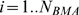 samples from the posterior density 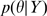. For each sample, 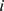, and subject 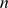 we first select a model as follows. For RFX we draw from
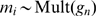
(30)where the 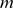
th element of the vector 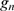 is the posterior model probability for subject 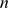, 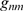 (we will use the expected values from equation 14). For FFX the model probabilities are the same for all subjects and we draw from
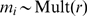
(31)where 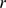 is the 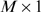 vector of posterior model probabilities with 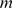
th element equal to 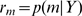. For each subject one then draws a single parameter vector, 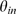 from the subject and model specific posterior
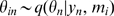
(32)These 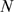 samples can then be averaged to produce a single sample
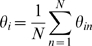
(33)One then generates another sample by repeating steps 30/31, 32 and 33. The 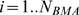 samples then provide a sample-based representation of the posterior density 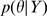 from which the usual posterior means and exceedance probabilities can be derived. Model averaging can also be restricted to be within-subject (using equations 30/31 and 32 only). Summary statistics from the resulting within-subject densities can then be entered into standard random effects inference (eg using t-tests) [19].

For any given parameter, some models assume that the parameter is zero. Other models allow it to be non-zero and its value is estimated. The posterior densities from equation 27 will therefore include a delta function at zero, the height of which corresponds to the posterior probability mass of models which assume that the parameter is zero. For the applications in this paper, the posterior densities from equation 27 will therefore correspond to a mixture of delta functions and Gaussians because 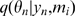 for DCMs have a Gaussian form. This is reminiscent of the model selection priors used in [39] but in our case we have posterior densities.

## Results

We illustrate the methods using neuroimaging data from a previously published study on the cortical dynamics of intelligible speech [17]. This study applied dynamic causal modelling of fMRI responses to investigate activity among three key multimodal regions: the left posterior and anterior superior temporal sulcus (subsequently referred to as regions P and A respectively) and pars orbitalis of the inferior frontal gyrus (region F). The aim of the study was to see how connections among regions depended on whether the auditory input was intelligible speech or time-reversed speech. Full details of the experimental paradigm and imaging parameters are available in [17].

An example DCM is shown in figure 1. Other models varied as to which regions received direct input and which connections could be modulated by ‘speech intelligibility’. Given that each intrinsic connection can be either modulated or not, there are 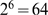 possible patterns of modulatory connections. Given that the auditory stimulus is either a direct input to a region or is not there are 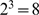 possible patterns of input connectivity. But we discount models without any input so this leaves 7 input patterns. The 64 modulatory patterns were then crossed with the 7 input patterns producing a total of 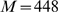 different models. These models were fitted to data from a total of 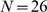 subjects (see [17] for details). Overall 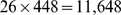 DCMs were fitted. The next two sections focus on family level inference. As this is a methodological paper we present results using both an FFX and RFX approach (ordinarily one would use either FFX or RFX alone).

### Input Regions

Our first family level inference concerns the pattern of input connectivity. To this end we assign each of the 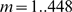 models to one of 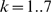 input pattern families. These are family A (models 1 to 64), F (65 to 128), P (129 to 192), AF (193 to 256), PA (257 to 320), PF (321 to 384) and PAF (285 to 448). Family PA, for example, has auditory inputs to both region P and A.

**Figure 2:**
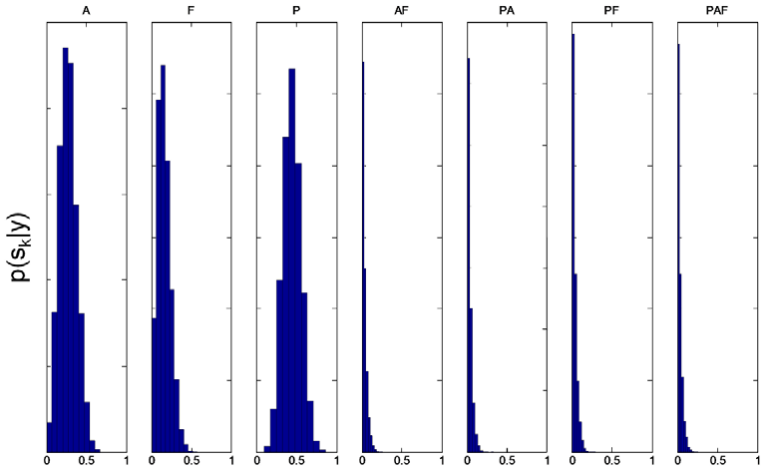
RFX posterior densities for input families. The histograms show 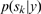 versus 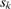 for the 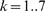 input families. Input family ‘P’ has the highest posterior expected probability 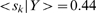. See Table 1 for other posterior expectations.

The first two numerical columns of Table 1 show the posterior family probabilities from an FFX analysis computed using equation 21. These are overwhelmingly in support of models in which region P alone receives auditory input (alternative probability 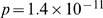). The last two columns in Table 1 show the corresponding posterior expectations and exceedance probabilities from an RFX analysis computed using equation 25. The conclusions from RFX analysis are less clear cut. But we can say, with high confidence (total exceedance probability, 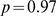) that either region A alone or region P alone receives auditory input. Out of these two possibilities it is much more likely that region P alone receives auditory input (exceedance probability 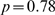) rather than region A (exceedance probability 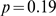). Figure 2 shows the posterior distributions 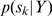, from an RFX analysis, for each of the model families.

**Table 1:** Inference over input families.

Input	FFX		RFX	
	Posterior 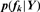	Log Posterior 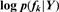	Expected 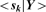	exceedance 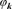
A	0.00	−25.33	0.27	0.19
F	0.00	−55.08	0.16	0.03
P	1.00	0.00	0.44	0.78
AF	0.00	−86.97	0.03	0.00
PA	0.00	−61.70	0.03	0.00
PF	0.00	−68.59	0.03	0.00
PAF	0.00	−134.67	0.03	0.00

All values are tabulated to two decimal places (dp). For an FFX inference, the alternative probability for input family 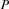 is 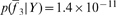. The expected and exceedance probabilities for RFX were computed from the posterior densities shown in Figure 2. For RFX inference the total exceedance probability that either region A alone or region P alone receives auditory input is 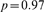.

### Forward versus Backward

Having established that auditory input most likely enters region 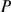 we now turn to a family level inference regarding modulatory structure. For this inference we restrict our set of candidate models, 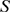, to the 64 models receiving input to region 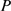. We then assign each of these models to one of 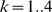 modulatory families. These were specified by first defining a hierarchy with region P at the bottom, A in the middle and F at the top; in accordance with recent studies that tend to place F above A in the language hierarchy [40]. For each structure we then counted the number of forward, 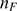, and backward, 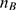, connections and defined the following families: predominantly forward (F, 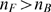), predominantly backward (B, 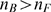), balanced (BAL, 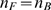), or None.

**Figure 3:**
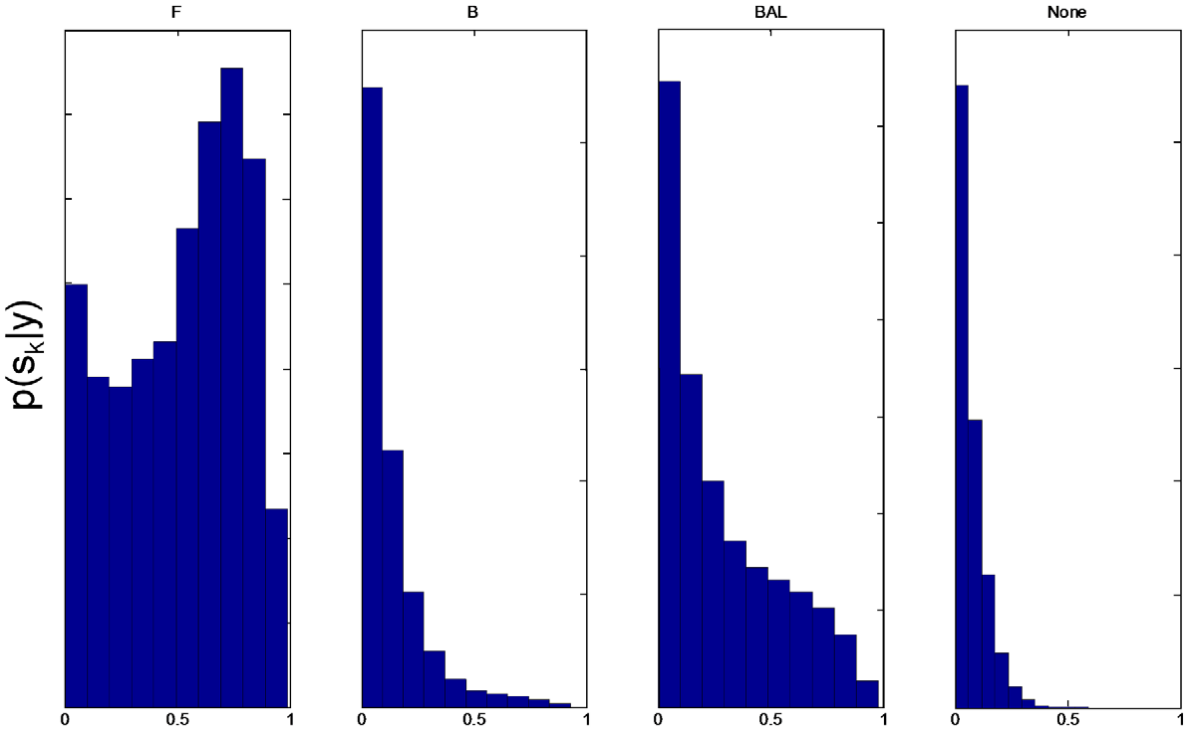
RFX Posterior densities for modulatory families. The histograms show 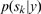 versus 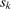 for the 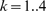 modulatory families. Modulatory family ‘F’ has the highest posterior expected probability 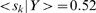. See Table 2 for other posterior expectations.

The first two numerical columns of Table 2 show the posterior family probabilities from an FFX analysis. We can say, with high confidence (total posterior probability, 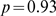) that 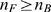. The last two columns in Table 2 show the posterior expectations and exceedance probabilities from an RFX analysis. These were computed from the posterior densities shown in Figure 3. The conclusions we draw, in this case, are identical to those from the FFX analysis. That is, we can say, with high confidence (total exceedance probability, 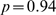) that 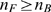.

**Table 2:** Inference over modulatory families.

Modulation	FFX		RFX	
	Posterior 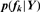	Log Posterior 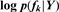	Expected 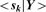	exceedance 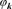
Forward, 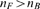	0.64	−0.44	0.52	0.66
Backard, 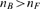	0.07	−2.71	0.13	0.06
Balanced, 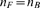	0.29	−1.22	0.28	0.28
None	0.00	−38.37	0.07	0.00

All values are tabulated to two decimal places (dp).

### Relating Family and Model Levels

**Figure 4:**
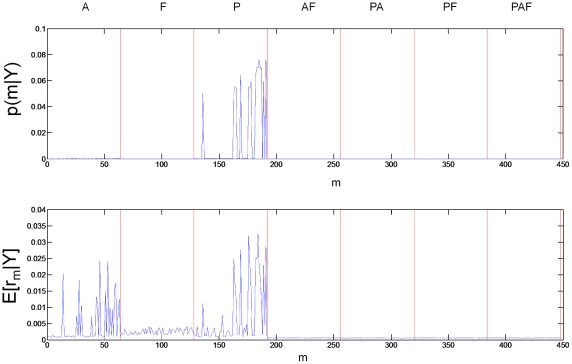
Model level inference for input families. For FFX (top panel) the figure shows that models in the P family have by far the greatest posterior probability mass. For RFX (bottom panel) models in both A and P families have high posterior expected probability, although the probability mass for P dominates.

**Figure 5:**
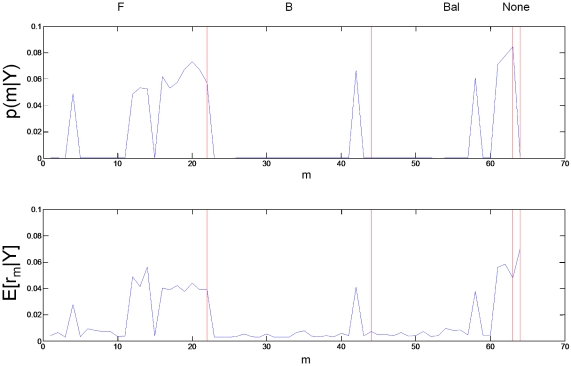
Model level inference for modulatory families. For FFX (top panel) the figure shows that models in the F and BAL families have most probability mass. The expected posteriors from the RFX inference show a similar pattern (bottom panel). The ordering of models in this figure is not the same as the ordering of P models in figure 4.

Family level posteriors are related to model level posteriors via summation over family members according to equation 21 for FFX and equation 22 for RFX. Figure 4 shows the how the posterior probabilities over input families break down into posterior probabilities for individual models. Figure 5 shows the same for the modulatory families.

**Figure 6:**
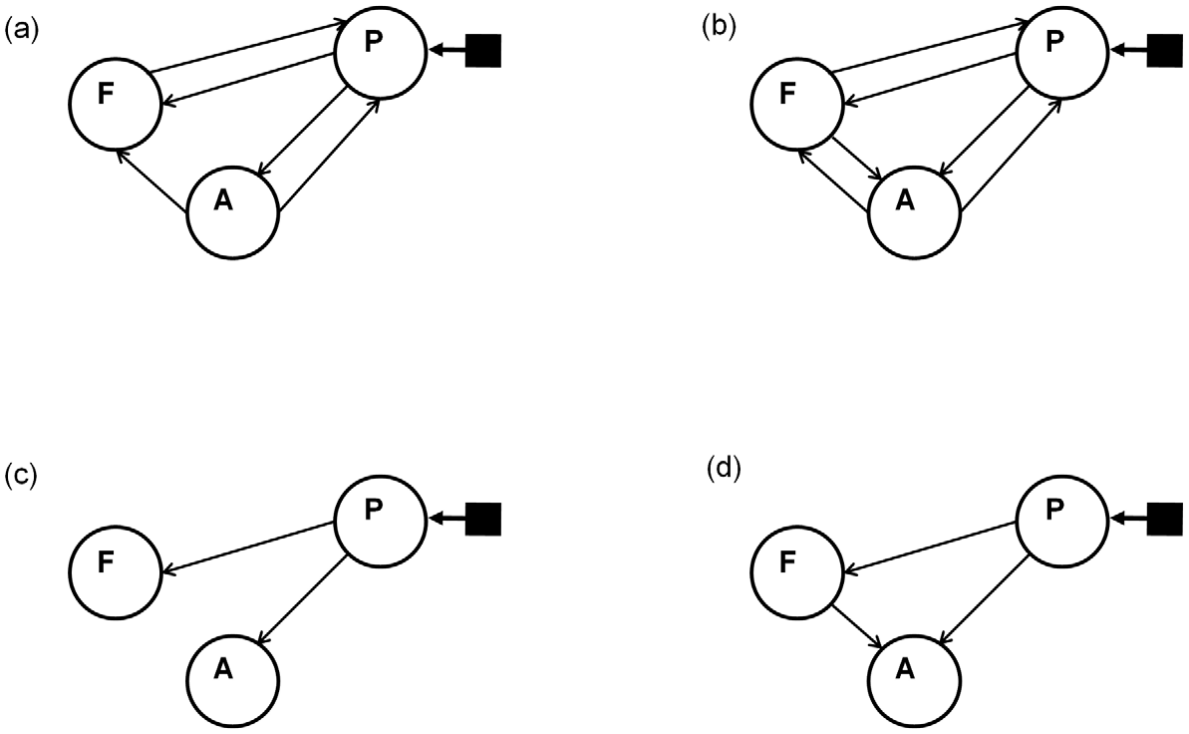
Likely models. The figure shows the input (filled square and solid arrow) and modulatory connectivity (solid arrows) stuctures for four models in Occam's window (assessed using FFX). Note that all models also have full endogenous connectivity (not shown). These four models are (a) model 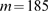 with 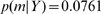, rank = 1, (b) model 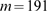 with 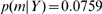, rank = 2, (c) model 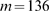 with 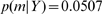, rank = 15 and (d) model 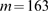 with 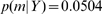, rank = 16. All models have auditory input entering region P.

The maximum posterior model for the input family inference is model number 185 having posterior probability 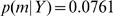. Given that all families have the same number of members, the model priors are uniform, so the maximum posterior model is also the one with highest aggregate model evidence. This model has input to region P and modulatory connections as shown in Figure 6(a).

The model evidence for the DCMs fitted in this paper was computed using the free energy approximation. This is to be contrasted with previous work in which (the most conservative of) AIC and BIC was used [17]. One notable difference arising from this distinction is that the top-ranked models in [17] contained significantly fewer connections than those in this paper (one sample t-test, 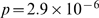). The top 10 models in [17] contained an average 2.4 modulatory connections whereas those in this paper contained an average of 4.5. This difference reflects the fact that the AIC/BIC approximation to the log evidence penalizes models for each additional connection (parameter) without considering interdependencies or covariances amongst parameters, whereas the free energy approximation takes such dependencies into account.

### Model Averaging

We now follow up the family-level inferences about input connections with Bayesian model averaging. As previously discussed, this is especially useful when the posterior model density is not sharply peaked, as is the case here (see Figure 4. All of the averaging results in this paper are obtained with an Occam's window defined using a minimal posterior odds ratio of 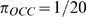.

**Figure 7:**
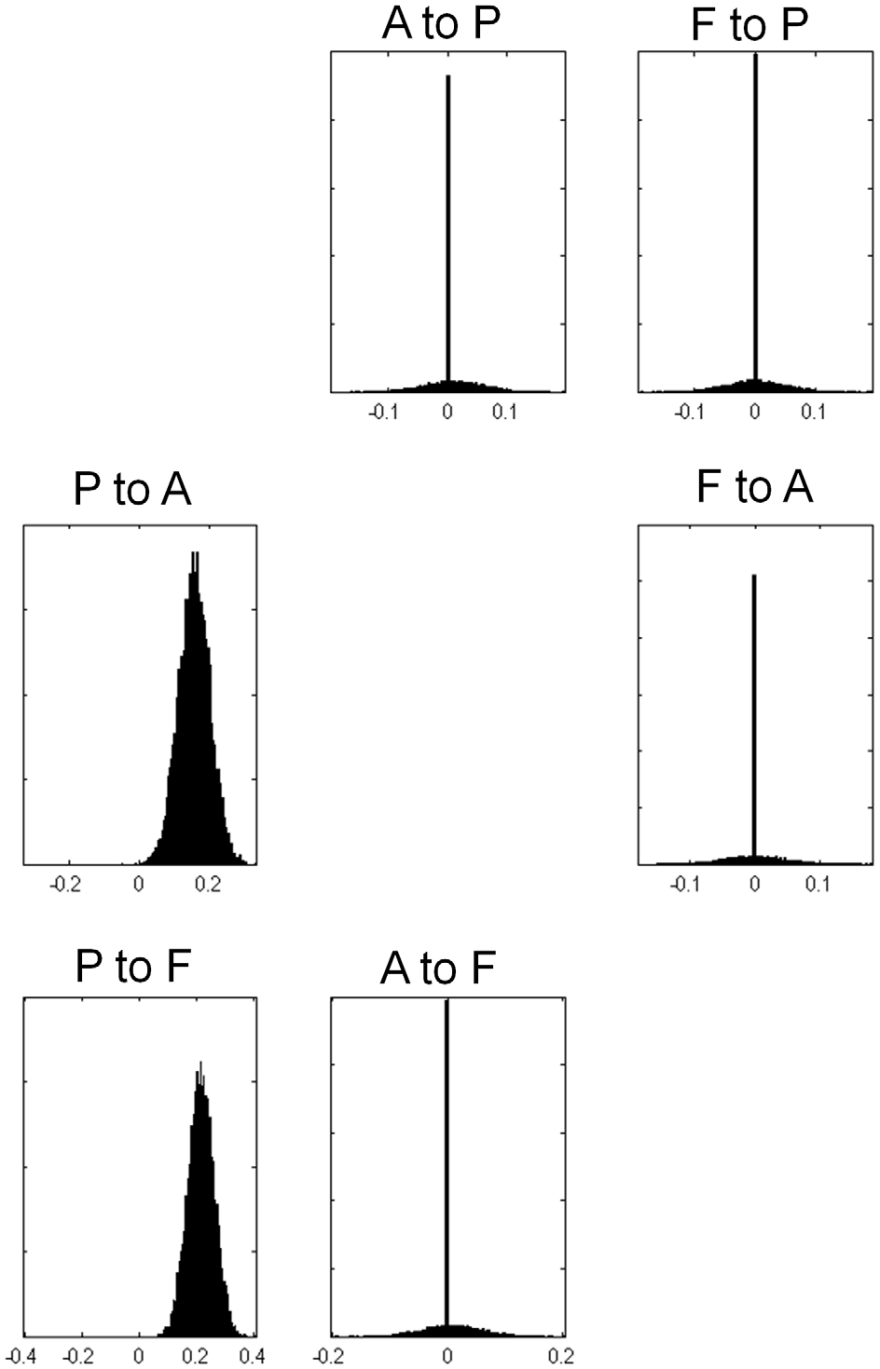
Average Modulatory Connections from FFX for input family P. The figures show the posterior densities of average network parameters from fixed effects Bayesian model averaging for the modulatory connections. Only forward connections from P to A and from P to F are modulated by speech intelligibility.

For FFX inference the input was inferred to enter region P only. We therefore restrict the averaging to those 64 models in family P. This produces 16 models in Occam's window (itself indicating that the posterior is not sharply peaked). The worst one is 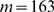 with 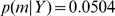. The posterior odds of the best relative to the worst is only 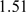 (the largest it could be is 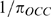), meaning these models are not significantly better than one another. Four of the models in Occam's window are shown in Figure 6. Figure 7 shows the posterior densities of average modulatory connections (averaging over models and subjects). The height of the delta functions in these histograms correspond to the total posterior probability mass of models which assume that the connection is zero.

**Figure 8:**
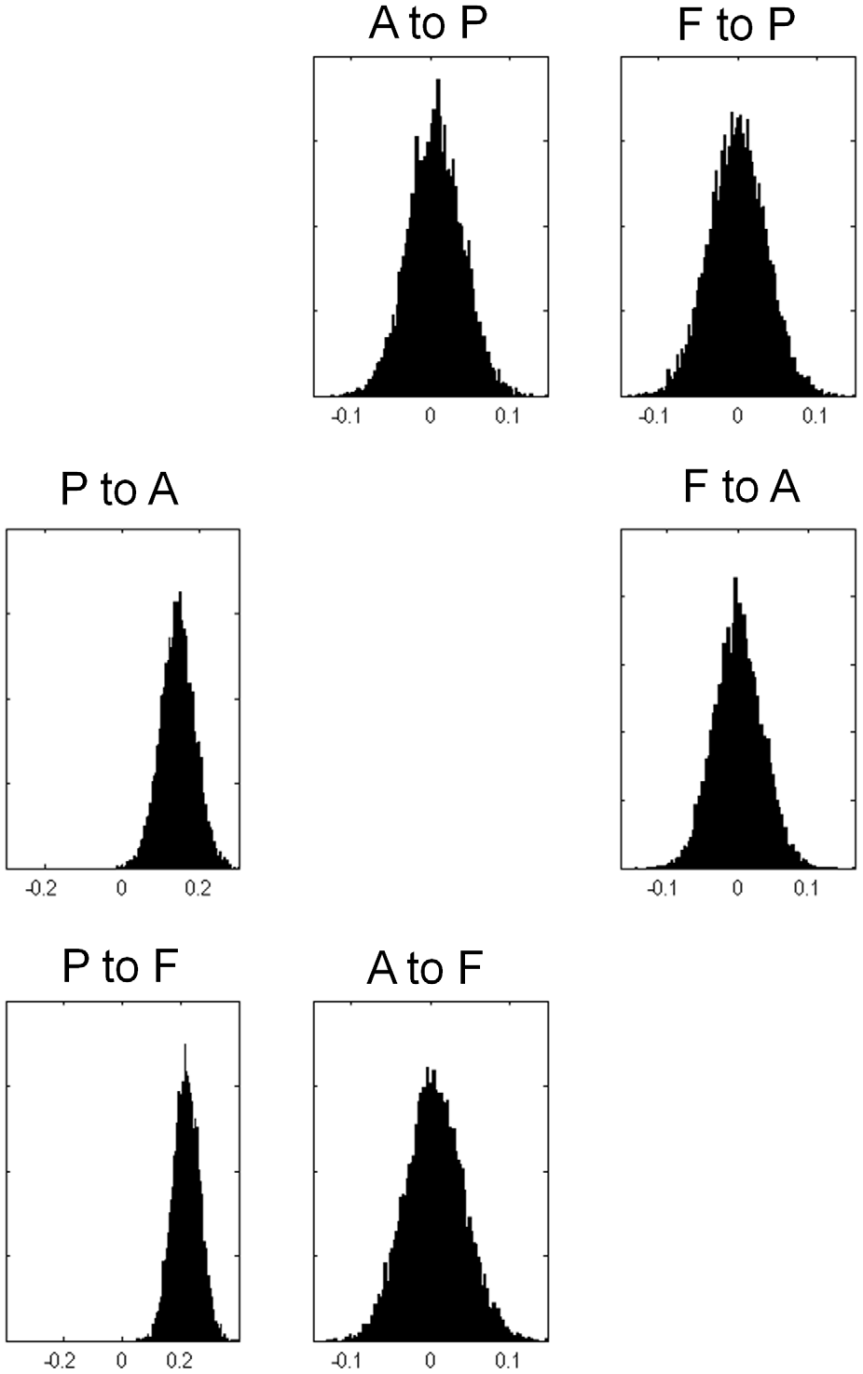
Average Modulatory Connections from RFX for input family P. The figures show the posterior densities of average network parameters from random effects Bayesian model averaging for the modulatory connections. Only forward connections from P to A and from P to F are modulated by speech intelligibility.

For RFX inference the input was inferred to most likely enter region P alone (posterior exceedance probability, 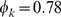). In the RFX model averaging the Occam's windowing procedure was specific to each subject, thus each subject can have a different number of models in Occam's window. For the input model P family there were an average of 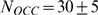 models in Occam's window and Figure 8 shows the posterior densities of the average modulatory connections (averaging over models and subjects). Both the RFX and FFX model averages within family P show that only connections from P to A, and from P to F, are facilitated by speech intelligibility.

## Discussion

This paper has investigated the formal comparison of models using Bayesian model evidence. Previous application of the method in the biological sciences has focussed on model selection in which one first selects the model with the highest evidence and then makes inferences based on the parameters of that model. We have shown that this ‘best model’ approach, though useful when the number of models is small, can become brittle if there are a large number of models, and if different subjects use different models.

To overcome this shortcoming we have proposed the combination of two further approaches (i) family level inference and (ii) Bayesian model averaging within families. Family level inference removes uncertainty about aspects of model structure other than the characteristic one is interested in. Bayesian model averaging can then be used to provide a summary measure of likely parameter values for each family.

We have applied these approaches to neuroimaging data, specifically a DCM study of auditory word processing using fMRI. Our results indicate that spoken words most likely stimulate a region in posterior STS and that if the word is intelligible connections are strengthened both to anterior STS and an inferior frontal region. These conclusions were drawn based on family level inference and Bayesian model averaging.

The model evidence for the DCMs fitted in this paper was computed using the free energy approximation whereas previous work used (the most conservative of) AIC and BIC [17]. This resulted in the highly ranked models containing significantly more connections than in the previous study. This is due to a bias in the AIC/BIC criterion which leads to overly simple models being selected. Previous work in graphical models favours the free energy approach over BIC [6] and work on biochemical models finds AIS to be the best of the more computationally expensive sampling methods. The relative merits of the different model selection criteria, as applied to brain imaging models and data, will be addressed in a future publication. The family level inference procedures described in this paper can be applied whatever method is used for estimating the model evidence.

Interestingly, the use of BMA produced an average network structure with speech input to region P, and modulatory connections from P to A and from P to F. This is exactly the winning model from earlier work [17] (based on AIC/BIC approximation of model evidence). It is not, however, the best model as indicated by the free energy. The model with the highest free energy (see figure 6(a)) does not, however, have significantly higher evidence than the second best model, or indeed, any model in Occam's window. This indicates that in the particular example we have studied the use of Bayes factors or posterior odds ratios would be inconclusive, whereas clear conclusions can be drawn from family level inference.

This paper has also introduced a Gibbs sampling method for RFX model level inference when the number of models is large. This sampling method should be preferred to the previously suggested VB method [19] when the number of models exceeds the number of subjects (ie. 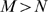). We do emphasise, however, that for RFX model level inferences involving a small number of models (as in previous work [19]) the VB approach is perfectly valid, and is indeed the preferred approach because it is faster.

The issue of family versus model level inference is orthogonal to the issue of random versus fixed effects analysis. The same critera re. FFX versus RFX apply at the family level as at the model level. For the data in this paper one might use RFX analysis as auditory word processing is part of the high level language system and one expect might expect differences in the neuronal instantiation (eg. lateralisation). If the issue remains unclear one could adopt a more pragmatic approach by first implementing a FFX analysis, and if there appear to be outlying subjects, then one could follow this up with an RFX analysis.

Family level inferences under FFX assumptions are simple to implement. Families with (the same and) different numbers of models are accommodated by setting model priors using equation 20, model posteriors are computed using equation 7, and family level posteriors using equation 21. This is a simple non-iterative procedure. Family level inferences under RFX assumptions are more subtle and have been the main focus of this paper. Families with (equal and) unequal numbers of models are accommodated using the model priors in equation 24, model posteriors are computed using an iterative Gibbs sampling procedure, and family level posteriors are computed using equation 22. We envisage that family level inference under RFX assumptions will be particularly useful in neuroimaging studies of high level cognition or for clinical groups where there is a high degree of intersubject variability. Where subjects can be clearly divided into two or more groups on behavioural or other grounds (e.g. patients and controls), then it would be correct to group the models accordingly, and proceed with a between group analysis on selected parameters of the averaged models.

Finally, we comment on the broader issue of comparison of discrete models (the ‘Discrete’ approach adopted in this work) versus a hierarchical approach embodying Automatic Relevance Determination (ARD) in which irrelevant connections are ‘switched off’ during model fitting [41] (for the case of DCMs the ARD approach is currently hypothetical as no such algorithm has yet been implemented). The ARD approach provides an estimate of the marginal density 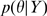 directly without recourse to Bayesian model averaging. The Discrete approach allows for quantitative family-level inferences about issues such as whether processing is serial or parallel, linear or nonlinear. Additionally, Bayesian Model Averaging can be used with the Discrete approach to provide estimates of the marginal density 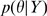. Overall, the ARD approach is probably the preffered method if one is solely interested in the marginal density over parameters, because it will likely be faster. If one is additionally interested in quantitative family-level inference then the Discrete approach would be the method of choice.

We expect that the comparison of model families will prove useful for a range of model comparison applications in biology, from connectivity models of brain imaging data, to behavioural models of learning and decision making, and dynamical models in molecular biology.

## Supporting Information

Text S1 Supplementary Information
(0.08 MB PDF)
