# Supplementary material for: Comparing Families of Dynamic Causal Models
Source: PLoS Comput Biol. 2010 Mar 12;6(3):e1000709. doi: 10.1371/journal.pcbi.1000709 (PMC2837394; doi:10.1371/journal.pcbi.1000709)
Supplement: Text S1 — Supplementary Information (0.08 MB PDF) [file pcbi.1000709.s001.pdf]

Comparing Families of Dynamic Causal Models: Supplementary Material

Will D. Penny[1,δ], Klaas E. Stephan[1,2], Jean Daunizeau[1,2], Maria J. Rosa [1], Karl J. Friston[1], Tom M. Schofield[1] and Alex P. Leff[1]

Wellcome Trust Centre for Neuroimaging[1], University College, London WC1N 3BG, UK.

Branco-Weiss Laboratory for Social and Neural Systems Research[2] Empirical Research in Economics, University of Zurich, Switzerland.

[δ]Corresponding Author: *w.penny@fil.ion.ucl.ac.uk*

December 21, 2009

VB for RFX inference

In previous work we have proposed a VB algorithm for RFX inference over models. This algorithm (see equation 9 in [2]) makes use of the relation

$$\int q(r_m) \log r_m dr_m = \psi(\alpha_m) - \psi(\sum_j \alpha_j) \tag{1}$$

where $q(r) = \mathsf{Dir}(\alpha)$ and $\psi()$ is the digamma function [1]. We can also evaluate the above expression using a sample based approximation

$$\int q(r_m) \log r_m dr_m \approx \frac{1}{S} \sum_{i=1}^{S} \log r_m^{(i)} \tag{2}$$

where $r_m^{(i)}$ are samples from $q(r_m)$. Setting $\alpha_m = 1/M$, our simulations show that the above two expressions begin to diverge for $M > 8$ with equation 1 producing excessively negative values. This occurs for two different implementations of the ψ function (the MATLAB R2009a routine `psi.m`, and the `fdigamma.m` function from Matlab Central file exchange, `http://www.mathworks.com/matlabcentral/fileexchange/`). We therefore conclude that equation 1 does not hold for $\alpha_m \leq 1/8$. This renders the VB approximation inaccurate if the number of models is large.

Software Note

The algorithms described in this note have been incorporated into the current version of the SPM software (SPM8, http://www.fil.ion.ucl.ac.uk/spm/) using the functions `spm_BMS_gibbs.m` and `spm_compare_families.m`. The functionality of these algorithms can be accessed via the batch user interface (Select 'Tasks','SPM(interactive)','Stats','BMS:DCM').

References

[1] W. H. Press, S.A. Teukolsky, W.T. Vetterling, and B.V.P. Flannery. *Numerical Recipes in C.* Cambridge, 1992.

[2] K. Stephan, W. Penny, J. Daunizeau, R. J Moran, and K. J. Friston. Bayesian model selection for group studies. *Neuroimage*, 46(4):1004–17, 2009.
